# Is Buying Local Less Expensive? Debunking a Myth—Assessing the Price Competitiveness of Local Food Products in Canada

**DOI:** 10.3390/foods11142059

**Published:** 2022-07-12

**Authors:** Sylvain Charlebois, Amy Hill, Melanie Morrison, Janele Vezeau, Janet Music, Kydra Mayhew

**Affiliations:** 1 Agri-Food Analytics Lab, Dalhousie University, Halifax, NS B3H 4R2, Canada; sylvain.charlebois@dal.ca (S.C.); amy.hill@dal.ca (A.H.); kydra.mayhew@dal.ca (K.M.); 2 College of Arts and Science, University of Saskatchewan, Saskatoon, SK S7N 5A2, Canada; melanie.morrison@usask.ca; 3 Canadian Agri-Food Foresight Institute, Halifax, NS B2X 3T5, Canada; janele.vezeau@cafi-icpa.ca

**Keywords:** local food, affordability, Canada, consumer behavior, food choice

## Abstract

It is well known that many consumers believe local foods are more expensive than comparative products coming from other markets. The aim of this study was to measure the price competitiveness of products certified by the Aliments du Québec program, a well-known program in the Canadian province of Quebec. Using machine-learning, artificial intelligence and targeted data mining, the report identifies local products and comparator products, to consider whether locally certified products are more expensive than comparative products coming from outside Quebec. Uncertified products used as comparative products come from anywhere around the world, outside of the province of Quebec. For this study, a total of more than 350,000 discrete price data points were analyzed in the Winter 2022. Local product prices were examined relative to the prices of comparator products. In total, there were 48 subcategories considered. In 70.83% of the subcategories, the local product was either as expensive (similar price) or less expensive than the comparator product. Results challenge the popular belief that local food products are often more expensive. This study also provides limitation and future research paths.

## 1. Introduction

The cost of living is rising significantly for Canadian households. From January 2021 to January 2022, in the Canadian province of Quebec, inflation for shelter rose by 4.8%, energy prices rose by 20.1% and service price inflation rose by 3.6%. In the same time period, food price inflation in Quebec rose above 9% [1]. In the food context, Quebec consumers are contending with “shelflation”, when supply chain inefficiencies impact the shelf-life of food products at retail [2,3,4], and there are supply chain disruptions from labor shortages, the pandemic, extreme weather and global conflicts [5,6]. One report forecasted that food prices will continue to rise in 2022 by somewhere between 5% and 7%, depending on the category. According to Statistics Canada, in 2019 the average annual household food expenditure in Quebec was $9847—prior to these significant inflationary increases [7,8]. With the cost of goods and services continuing to rise in the province, and the employment rate still recovering to pre-pandemic levels, Quebecers are considering ways to reduce food expenditure. In doing so, consumers may be tempted to stay away from local foods as they are often perceived as being more expensive [9].

In Canada, the definition of a local food product will vary, and will depend on perspectives [10,11]. Some might consider local food as a product produced or processed within their community, or within a certain physical distance, whereas other consumers may consider product as being local within a jurisdiction, say a province or country. An Ontario study found that: “most Ontario consumers perceive local foods as being from that region or county [12,13]”. A consumer’s definition of local might also depend more so on the size of the producer, rather than the location of the producer.

While there may be no single definition of local in the context of food production, there are some common values and themes, which underpin consumer preferences for local foods. Generally, consumers value local food for several reasons, including: reduced environmental cost and emissions, due to shorter transportation in the supply chain; to support producers within their communities and build their local economy; and concerns regarding food safety [14].

Given the current economic climate, and disruptions to supply chains across the world, this study explores the notion of whether local products are competitively priced. The study focuses on Quebec, where a well-known and recognized certification program for local products exists and has been implemented for years. As such, this report operates on the following definition of local: a product whose “main production, processing and packaging activities are carried out in Quebec, [with a] valid Quebec Enterprise Number (NEQ)”. The unique nature of Aliments du Québec allowed this study to rely on the implicit consensus of what can be considered as a local product, and what is not. Using machine learning, artificial intelligence and data mining, this report examines thousands of discrete price data points for local and comparator products to consider how competitive local products are compared to other products not certified as local in the province of Quebec. We first explain the methodology, before presenting results. An analysis for all subcategories is presented, followed by concluding remarks.

### 1.1. Local Food

The eating local paradigm in Canada often suggests that locally sourced fruit and vegetables are predominantly the one category where localization is possible. Although local foods can be found in all sections of the grocery store, due to the shortened growing season in Canada, the term local food is often associated with fruit and vegetables. Concern for the environmental implications of a potentially unsustainable global food supply system has led to a call for shorter supply chains, and a renewed interest in community food supply [15,16,17]. Furthermore, many consumers have a distrust of food that they perceive may be genetically modified [18], and therefore turn to ‘local food’ to avoid such produce. In Canada, the local food movement boomed in the 2000s [19]. Community gardens and local farmers’ markets existed prior, but the social activism behind local food boosted its popularity and increased its reach within communities [19,20,21]. Ultimately, local food allows consumers to have greater control over food production [22,23], which is associated with small, rural producers of local produce [24].

Accessibility and cost are also major contributing factors for consumers purchasing locally produced food. Supermarkets and similar retailers receive food through supply chain intermediaries, increasing the time between production and consumption, whereas purchasing directly from local vendors allows for direct distribution of fresher items [25]. Due to the ease of accessibility and quality of products, there is a widespread perception that local products are significantly higher in price than imported foods [9]. One such reasoning behind this could be that local food producers tend to promote the “ethical” nature of production of goods, emphasizing how sustainability, labor and wages factor into the cost of products [26]. However, research has produced mixed results in Canada. Noseworthy et al. compared pricing of local products in grocery stores across Nova Scotia, finding that locally produced products were less expensive than mass produced products, in 75% of stores [27].

Community based health education has been proven in global situations to help promote change in dietary habits of a population [28]. With the rise of these health education programs, and the sustainable food movement, consumers’ desire to eat local has increased. However, in comparison with the global integrated food system, an over-reliance on local food structures could lead to depletion, and eventually failure [29]. It is argued that an over-reliance on local food structures in areas where there is not already a strong agriculture system in place, could deplete resources even further [29].

Modern agriculture is a resource-laden industry. Along with land, water and financial resources, producing food requires technical skills not possessed by many [30]. Partial food autonomy within urban communities, is therefore achieved by growing fruit and vegetables in community and household gardens [31,32]. Gardeners cite responsible food production and food security among the reasons for growing fruit and vegetables [17,33]. Those consumers that spent their developing years in a family that produced fruit and vegetables, either in a household garden, rural region or farm, are more likely to cultivate their own food [33].

### 1.2. Perishable Food

Recent research has examined “Just-In-Time” practices for supply chain planning—and more specifically the tension this causes on global food systems when it comes to product availability and quality [34]. Food rot and degradation during transit can result in market loss and value depreciation when there are supply chain constraints [35]. Although supply chain disruptions can result in the waste of shelf-stable products, fresh products that require cold chain capabilities are more susceptible to perishability while in transport [36], as cold chain technologies allow for longer periods of preservation and shelf-life during transportation [37,38,39]. Depending on whether a product needs to be kept frozen or chilled, transportation with cold chain capabilities needs to have availability for temperatures ranging from −25 °C to 8 °C.

There are many factors that can affect supply chain performance that need to be considered. In Canada, seasonality prohibits the growth of fresh products year-round. This results in the need to import products from other countries, and due to the size of the country products are often stuck in long periods of transit before arrival at a retailer [40]. Unforeseen natural calamities, such as harsh weather conditions or geohazards, are likely to cause delays and can impact product quality [41]. Although natural circumstances can cause unavoidable disruptions to supply chain mechanisms, mistakes or human induced errors such as labor shortages or mechanical failures, are preventable [42]. Due to the potential for product perishability based on the supply chain disruption, it is crucial that retailers are assessing the quality of incoming products, so that we can better understand the effects of supply chain delays on fresh product conditions to avoid “shelflation”.

## 2. Materials and Methods

Methodological choices were not trivial for this study. Few studies have measured the price competitiveness of local foods in all food categories. With machine learning and precise data mining capabilities, it is now possible to compare thousands of retail prices at given periods, without physically visiting stores. Comparing prices could have been carried out in many ways. Parameters of a comparative analysis was implemented by working with AdQ (Aliments du Québec).

Local products were selected from those products in Quebec certified by Aliments du Quebec (‘AdQ’), a provincially funded non-profit organization [43]. To be certified by AdQ, a product is reviewed to ensure that the “main production, processing and packaging activities [are] carried out in Quebec, [with a] valid Quebec Enterprise Number (NEQ) [44]. The verification process is based on provincial and federal legislation to corroborate “ingredients, their origin, and their availability in Quebec”, while also ensuring that the main processing and packaging occurs in Quebec. Once a product has been certified, it is still subject to monitoring and enforcement by AdQ compliance teams. The selection of AdQ-certified products was based on a review of government reports surrounding general food items which Canadians buy, provincial nutrition baskets, and Canada’s *Food Price Report*, alongside Statistics Canada’s food baskets [45,46]. These reports motivated how the food basket was structured. Once the AdQ products were selected, they were further researched to examine whether they met the characteristics of a generally nutritious shopping cart of goods, and to ensure coverage of items selected from all major grocery categories. The products selected were reviewed by AdQ to ensure that most people residing in Quebec would be able to purchase them.

A minimum of two similar products to the AdQ product were selected as comparator products. Food products were considered as comparator products when they were easily accessible to consumers, while not being certified by AdQ. Comparator products could have come from anywhere around the world. Machine-driven learning artificial intelligence through data mining was used to identify and select 10–15 comparison products of a similar size, content and brand quality to the AdQ product. Once recorded, a researcher reviewed and audited the selected comparator products. Once the products were selected, point-in-time price data were recorded for the AdQ-certified local products and non-certified comparative products, on six dates per the project’s mandate: 24 January 2022; 31 January 2022; 7 February 2022; 14 February 2022; 21 February 2022 and 28 February 2022. The prices were collected across six grocery retailers: IGA, Maxi, Metro, Provigo, Valu-Mart and Walmart. A total of more than 350,000 discrete price points were collected across the six dates. The chosen sampling design allowed us to avoid busy holiday seasons, and assess prices during a winter period.

We also had to make other methodological choices. In some instances, there was only one price available for a local or comparator product at a point in time across all retailers, so that price was used as the representative price for the product. In other instances, however, there were two or more prices available for the same product at different retailers on the same date. Where the product prices were the same across all retailers, that price was used. For example, on January 24th, the price of President’s Choice-Free Lean Ground Beef (454 g) was recorded at $9.00 at two different retailers, so the price used for comparison to the locally produced beef was the $9.00 price.

Where the prices recorded at different stores for a single product on the same date differed, the relative standard deviation was used to determine by how much the prices differed around the mean. Relative standard deviation is calculated as:

$$\frac{\sigma }{\mu }$$

σ=Population standard deciation 

μ=Population mean 

where:

$$\sigma =\sqrt{\frac{\sum {\left(x_{i}-\mu \right)}^{2}}{N}}$$

and:

$$\mu =\frac{1}{n}\overset{n}{\underset{i=1}{\sum }}x_{i}$$

The relative standard deviation was calculated using the population formulas for both standard deviation and the mean, rather than the sample formulas. In each case, where there is more than one price and the prices differ, these prices might be considered a sample of the overall data set. For example, the price of St-Justin Mineral Water (750 mL) was recorded at $1.79 and $1.99 on January 24th, but these two prices represent only two data points of many more price points recorded for the water—price points which may be considered to be the population. Generally, the sample standard deviation is calculated using (n-1) on the bottom of the equation, to account for any variance in the sample when using the output to make conclusions about the population. Here, there are only two or three prices in most cases, and the output is not being used to generalize about the data set, so the population formula is appropriate. The purpose of calculating the relative standard deviation is rather to evaluate when a price should be considered an outlier and discarded, instead of being used to create an average representative price.

We also had to eliminate outliers and extreme cases, so results were not skewed, which included some prices that were either much too low or much too high. Food products may be subject to loss leading, or could have been sold at a higher price that given week.

Where the relative standard deviation between two or more prices was 20% or less, the prices were averaged to create a representative product price. For example, on 24 January 2022, the price of St-Justin Mineral Water (750 mL) was recorded as $1.79 at one grocer and $1.99 at another grocer. The mean is calculated as follows:

$$\mu =\frac{1}{2}\left(1.99*1.79\right)=1.8$$

The standard deviation is calculated as follows:

$$\sigma =\sqrt{\frac{{\left(1.99-1.89\right)}^{2}+{\left(1.79-1.89\right)}^{2}}{2}}=0.10$$

The relative standard deviation is calculated, then, as:

$$\frac{\sigma }{\mu }=\frac{0.10}{1.89}=0.053$$

In this example, the relative standard deviation was 0.053 or 5.3% when multiplied by 100, so the two prices were averaged as the relative standard deviation between the two prices around the mean was less than 20%.

In cases where the relative standard deviation between the products was greater than 20%, expert judgement was used to determine whether the distribution of prices was representative of retail practices, such as loss-leading or a clearing of inventory. For example, on 24 January 2022, the price of Villas Cheese Tortellini (400 g) was recorded as $2.54 at one retailer and $4.49 at another retailer. In this example, the $2.54 price was discarded as an example of inventory clearing or loss leading, and the price of $4.49 was used as the local price to which the prices of other non-local products would be compared. The $2.54 price was not only lower than the $4.49 price, but when comparing the price to the overall data from different grocers across the different weeks, it is clearly an outlier.

Once any outliers were removed and any prices were averaged to create one single representative price per product per point-in-time, the local price was compared to the comparator product’s price, to determine the percentage change between the prices of the local product and the comparator product, using the following formula:

$$=\frac{(P_{\mathrm{Local}\;\mathrm{Product}-}P_{\mathrm{Comparator}\;\mathrm{Product})}}{P_{\mathrm{Comparator}\;\mathrm{Product}\;}}*100$$

If the output is negative from this formula, this means that the local product is less expensive, relative to the comparator product. Where the output is positive, the local product is more expensive, relative to the comparator product.

Five percentage-based measures were ultimately calculated from the data. The first three measures were:

**1.**  The proportion of times the local product was less expensive relative to the comparator products;
**2.**  Where the local product was more expensive on average, and by how much;
**3.**  Where the local product was less expensive on average, and by how much.

Finally, two additional measures were created to adjust for significant price fluctuations:

**4.**  An adjusted average for where the local product was relatively more expensive;
**5.**  An adjusted average for where the local produce was relatively less expensive.

The adjusted averages were calculated by removing any percentage price differences which were more than 20%, to account for any of the concerns noted above.

Finally, the competitiveness of the local products in relation to the comparator products was assessed, to allow the report to draw some broad conclusions based on the six-week point-in-time data. Where a local product category was, on average, between 0% and 30% of the time less expensive relative to the comparator groups, this was considered an advantage to the comparator product. Where a local product category was, on average, between 30% and 60% of the time less expensive relative to the comparator groups, this was considered neutral in terms of advantage to either the comparator or local product. Where a local product was less expensive relative to the comparator products more than 60% of the time, on average, this was considered an advantage to the local product. The adjusted relative percentage differences were also averaged across the categories, and included for context.

## 3. Results

The following results are organized in tables. Not all subcategories (48) are presented in this paper, for better readability. Each table includes six measures to consider (see Table 1 for outline instructions). The first measure is the number of comparator products, and the second measure is how often, relative to the comparator products, the local product was less expensive as a percentage (averaged across the six weeks of data).

The remaining four measures consider the price difference of the local product relative to the comparator products in percentage terms. The first two measures are relative differences without any adjustments.

The tables below also provide two relative percentage measures, which have been adjusted for large fluctuations in price, as mentioned in the Methodologies section. The measures in the final two columns are adjusted such that any relative price difference greater than 20% was removed, to create the average.

### 3.1. Milk & Milk Alternatives

Two different sizes of milks, 1 L (70, 71) and 2 L (68, 69), were considered below, alongside one soy beverage (72). More than 50% of the time, three of the four milks were, on average, less expensive than the comparator products (69–71) and by close to 20.00% when not adjusted. Where adjusted, the milks ranged from 2.43% (69) to 10.07% (70) less expensive. Milk 68 was less expensive 39.31% of the time, and by 16.04% or 9.55% when adjusted. Where milk 68 was more expensive, it was more expensive by 24.11% or 17.63 where adjusted. The local soy beverage was always less expensive than the other two comparator products, by 14.17% or 13.22% when adjusted.

Milk, where consumed, is purchased frequently by households and viewed as a homogenous good. The nature of milk, however, including the elements of animal welfare and climate emissions, may cause consumers to care more about the source of their milk. Quebec is a leading producer in dairy across Canada, with dairy being Quebec’s largest agricultural sector [47]. Dairy products are uniquely managed, by contrast, to the other products in this study. Dairy is managed under the supply management system, which includes import controls, pricing administration and quotas for domestic production [48]. Regardless, due to frequency and substitutability, in most cases it is likely that consumer demand for milk is more price elastic.

With regards to soy, while it does not possess the same complexities as the milk market, it is also purchased frequently by many households. Soy milks also appear to be somewhat homogenous, which may lead consumers to be more sensitive to price where there are many producers supplying a similar product. Quebec is one of the top producers of soy in Canada, which may explain in part why soy milk was always less expensive than its comparators [49]. (See Table 2).

### 3.2. Butter

Two local butters were examined in this section. Butter 75 was always more expensive than the 13 comparator butters, and was more expensive on average by 33.21% (7.78% where adjusted). Butter 76 was relatively less expensive 9.96% of the time by 14.38% on average (7.43% adjusted). Where butter 76 was less expensive, it was less expensive by 14.38% (13.22% adjusted).

Butter is generally perceived as a homogenous good and it is purchased frequently by many households. Substitutability and high purchase frequency may lead consumer demand to be elastic. Concerns regarding Canadian butter, however, surfaced in 2021, which may have led to some distrust by consumers in large national butter brands [50]. These concerns may have left consumers more wary of where their butter is being produced. (See Table 3).

### 3.3. Cheeses

Seven cheeses were examined. The first five cheeses (77–81) were relatively less expensive more than half of the time. Where these five cheeses (77–81) were more expensive, they ranged from 6.06% (77) to 30.46% (81) more expensive or 7.52% (81) to 14.53% (80) where adjusted. Where these five cheeses were relatively less expensive, they ranged from 7.52% (81) to 33.17% (77) less expensive. The final two cheeses (82,83) were less expensive 47.33% of the time. Cheeses 82 and 83 were less expensive 18.81% and 23.52%, or 7.66% and 14.53% where adjusted.

The market for cheese is varied and ranges from high product differentiation, in the case of more artisanal cheese, to less differentiation in the case of cheese consumed on a daily basis. What this means is that depending on the purpose of a cheese, whether to add to a daily sandwich or for a cheeseboard, demand elasticity will vary. If it is the case of a cheese which consumers are buying frequently to add to a sandwich, they are likely to be more price sensitive. In these instances, consumer willingness to pay may be lower for characteristics such as where a cheese was produced. By contrast, consumers purchasing a cheese for a special occasion may care more about other characteristics, and have a greater willingness to pay for a locally produced cheese (See Table 4).

### 3.4. Seafood

Three local salmon products were considered (87–89). The first seafood product (87) was relatively less expensive than the two comparator products 50% of the time by 38.93%. Where product (87) was more expensive, it was more expensive by 7.87%. Two salmon tartars were also considered (88,89). The first (88) was less expensive relative to the comparator products, 89% of the time by 6.69%. The second (89) tartar was relatively less expensive 8.25% of the time by 4.35%. Where tartar 89 was more expensive, it was more expensive by 4.35%.

Seafood is a sector in which consumers have more skepticism and concern about the product, due to concerns such as food fraud [51], the impact of marine environment pollution on product safety [52] and environmental damage from harvesting practices [53]. This awareness and concern may lead consumers to have a lower price elasticity in favor of locally produced goods. Seafood products in many households are purchased frequently, however, which may contribute to higher consumer responses to prices (See Table 5).

### 3.5. Meat

This section considers three meats: beef (90), chicken (90) and pork (91). The beef (90) was less expensive 50.00% of the time, relative to the 10 comparator ground beefs by on average 38.93%. Where the local beef was more expensive, it was, on average, more expensive by 7.87%. The local chicken (91) was less expensive relative to the nine comparator products 89.00% of the time by 6.69% on average. Finally, the pork (92) was less expensive by 16.02% (or 1.29% where adjusted) 53.40% of the time, relative to the four comparator pork products. Where the pork was more expensive, it was more expensive on average by 12.84% or 9.45% where adjusted.

Consumer demand and price responsiveness for meats is complex and affected by many factors, including perceptions of the local industry and consumer means. Consumers might be willing to pay more for locally produced meats where they may perceive the locally produced meats as being, for example, more promotive of animal welfare and food safety. Regardless, it is difficult to draw conclusions in this category because of these complexities, together with high prices and high frequencies with which many households purchase meats (See Table 6).

### 3.6. Apples

Four apple products were considered (103–106), each with a comparator product group of three. All of the apples were less expensive relative to the comparator products more than 70.00% of the time, by about 27.00% (no adjusted value available). Where the apples were more expensive, they were more expensive by about 12.00%.

The pandemic caused labor shortages across Canada for farmers, from which it is not clear that the sector has fully recovered. Regardless, Quebec is the second largest producer of apples in Canada, which may explain the somewhat reduced price of local apples [47]. Seasonality is also important to consider in apple prices, such that the apple harvesting season is outside of the time when the price data in this report were collected.

Apples are a product which most households buy frequently in larger quantities, so it is likely that they will be more responsive to price changes. Despite this, consumer willingness to pay for a locally produced product may be higher where they perceive the local product as being safer. Apples, for example, are generally eaten uncooked, so consumers may care more about how the apples were grown and produced. Consumers may perceive the local apples as being more amendable to those concerns. The market for apples, however, is also characterized by a large number of producers selling similar products. It is not clear that concerns and perceptions of local produce outweigh concerns about price and high purchase frequency (See Table 7).

### 3.7. Clamshell Salads, Fresh Herbs

Ten lettuce products were considered (107–116). Four of the ten products were less expensive relative to the comparator products more than 50.00% of the time (109, 111, 112, 116). Where these products were less expensive, they were less expensive by about 10.00% (109, 111, 112, 116). The remaining six products were less expensive relative to the comparator products less than half of the time (107, 108, 110, 113–115). Where data were available, it shows that these products were relatively less expensive by about 17%. Where the remaining six products were more expensive, they ranged from 12.00% (110) to 101.85% (108) or 4.33% (107) to 12.00% (110) where adjusted.

Fresh lettuce and herbs are purchased frequently and therefore consumers may be more sensitive to price. For example, consumers may not be willing to pay an additional 9.75% for arugula (107). There are often substitutes in the market for lettuce, though not as many as in other product markets, so the effects of some substitutes on consumer purchasing decisions is unclear. High frequency and some substitutes may result in a more elastic consumer demand (See Table 8).

### 3.8. Bread

Two types of local breads were examined in this category: white or whole wheat breads (118–125, 127), and raisin bread or bagels (126, 128–129). The first set of local breads (118–125, 127) appear to be less expensive depending on brand: 118–121 were all relatively less expensive more than three quarters or 75.00% of the time, by anywhere from 3.99% (120) to 26.10% (121). Where adjusted, the range for breads 118–121 was 3.99% (120) to 18.35% (121). Breads 122–125 were less expensive relative to the comparator group 15.00% of the time or less, by 2.98% (122) to 4.77% (123). Where the breads were more expensive, they were more expensive by, on average, 26% or, on average, between 1.69% (123) and 6.29% (124) where adjusted.

The local raisin bread products (126, 128–129) were all less expensive relative to the comparator group more than half of the time, by at least 26% or at least 13% where adjusted. These products were more expensive by a range of 14.97% (128) to 19.87% (129) or 6.46 (129) to 18.29 (128) where adjusted.

Households purchase bread frequently, and the market for bread is a market in which there are often many substitutes. As a result, consumer demand is likely more elastic. Cinnamon breads, however, are purchased less frequently compared to plain breads, so the consumer willingness to pay may be higher. Further, cinnamon products are more differentiated, which may further contribute to a more inelastic consumer demand (See Table 9).

### 3.9. Bagels

Two bagels were considered (134–135). The first bagel (134) was less expensive relative to the four comparators 38.20% of the time by, on average, 7.18%. Bagel 134 was, on average, more expensive relative to the comparators by 37.56% or 5.99% where adjusted. Bagel 135 was always more expensive than the comparator products, by an average of 82.75% (no adjusted values were available).

Bagels follow a similar trend to bread, as discussed above. Bagels are, in many cases, purchased frequently by households and there are often many substitutes. As a result, consumer demand is likely more elastic in this market. In this case, the unadjusted relative percentage difference between the local product and the comparators is high, which may further disadvantage the local product (See Table 10).

Overall results can be seen in Table 11.

## 4. Discussion

Assessing the price competitiveness of locally certified products was critical, given the economic context we are in. While many consumers believe local products to be more expensive, most of the time, the evidence this study provides challenges the dominant paradigm of local food affordability. In total, there were 48 subcategories considered. In 70.83% of the subcategories, the local product was either as competitive (similar prices) or more competitive than the comparator product.

While assessing the price competitiveness of local products in relation to comparator products is important, context matters. As discussed in the introduction, local products include many important characteristics, for which consumers may be willing to pay more for. That is, the nature of the product matters for the consumer when assessing whether the performance and magnitude of the relative percentage difference matters to them. Debunking the myth that local food products are more expensive is critical, especially in an inflationary environment.

Due to buying patterns and frequency, not all local product’s price competitiveness should be considered the same. Honey is a good example. Local honey performed relatively poorly compared to comparator honeys. The table above shows that local honey was always relatively more expensive, and by, on average, 11.85% where adjusted. Honey, however, compared to perhaps bread, is not something that most households are purchasing on a daily or even on a weekly basis. While local honey may have performed poorly, many consumers may feel that paying an additional 11.85% for the local honey they purchase less frequently is reasonable. That is, 11.85% may be well within their willingness to pay for honey.

By contrast, dairy products are purchased much more frequently in many households and are under the supply management system. The milks were considered neutral, meaning they were between 30% and 60% of the time relatively less expensive in relation to comparator products. Due to consumption of milk in many households, however, the magnitude of difference may matter more here, in contrast to the honey. On average, local milk was about 5% more expensive. Consumers might be more concerned about this difference depending on the frequency in which they purchase milk. That is, an increase in frequency of purchase likely means that the magnitude of percentage difference matters more for consumers.

Finally, it is interesting to examine the above data in terms of the layout of a traditional grocery store. At the center of a traditional store is generally the grocery and frozen departments. In this report’s imagined grocery department, seven local products were at a disadvantage to the comparator, nine were neutral, and six were at an advantage compared to the local product. In frozen, one product performed at an advantage to the comparator product and one at a level of neutral. There is no clear trend in the center of the store, except that there is a range in the products as to their performance. It is not possible to say that it is more or less expensive in the center of the store to purchase local, but rather it depends on the products.

At the perimeters of a traditional store are generally the so-called fresh departments: dairy, meat, seafood, produce, bakery and deli. In this report’s imagined seafood, meat, deli, bakery and produce departments, most of the products performed at either an advantage to the local product, or neutrally. In dairy, the results were less clear with three products performing at an advantage to the comparators, two were neutral and two were at an advantage to the local product. Similarly, to the center of the store, it is difficult to draw category-wide conclusions, except to say that in many of the fresh departments, a majority of the local products were either neutral or showed an advantage to the local product.

### Limitations

There are at least four limitations associated with this methodology. Firstly, there are some limitations regarding the use of relative standard deviation; that is, where the data set is small, the average may be skewed by one outlier. The relative standard deviation, however, is being used to help identify how big that spread is, and then to remove the outlier so that, ultimately, the percentage differences in prices will be less skewed.

Secondly, this methodology does not account for the differences in grocers. For example, certain grocers are known for their discounted prices, whereas others price higher for different reasons. What this means is that in some cases, a certain percentage of the price difference between, for example, the local price and a comparator product, may be attributed to the different pricing strategies of different grocers. For most weeks, however, only one grocer had a price available for either the local product or the comparator product, so this price was used as the representative price.

Thirdly, this methodology does not account for regional differences in pricing. Price data, where available, were collected for the individual grocery store locations across each banner. A total of 351,787 discrete price data points were recorded for the regional stores. For example, prices were collected for all the products at each individual store location in Quebec, under the Metro banner. For each banner, the standard deviation was calculated for each product across all regional stores. Almost all of the standard deviations were close to zero, which means there was limited variation, where there was variation, around the mean. While this methodology does not account for any regional pricing differences, those differences are small.

Finally, the point-in-time data collected is limited to six consecutive points in time. One of the dates, for example, February 14 is Valentine’s Day, which may have had some effect on the pricing of products. These dates are also all throughout winter, which may have resulted in further price effects for certain products. For example, on these dates, certain products were also not in season, such as salmon and apples. Any seasonal price variations were not accounted for by this methodology.

## 5. Conclusions

Given the certification program in the province of Quebec, a comparative analysis between local foods and products coming from elsewhere was easily achievable. The aim of the study was essentially to measure the price competitiveness of AdQ-certified products with comparators. Results of this unique study suggest that local food products are either as competitive, or more competitive than comparators, in most food categories. Such an outcome can obviously be seen as encouraging for local vendors.

A proper comparative analysis through data mining can debunk the myth that local foods are not competitively priced. However, given the study’s limitations and sample design, to suggest that local foods are always more competitive would be unfounded. More data mining during other seasons would be necessary to fully assess price competitiveness throughout the year. From a policy perspective, the idea may not be to assure that price competitiveness for local foods can be achieved for all food categories, but it is rather to know where local products stand, pricewise. It needs to be recognized that some local companies do want to set price points for local products which remain above market averages.

In light of these results, governments and industry ought to be encouraged to promote local foods, knowing food affordability is not automatically compromised. Local foods can be competitive, contrary to what most believe. The legacy of the certification program managed by AdQ may be that the program itself installs more discipline within the industry related to local foods. While local foods were heavily promoted, and generated more consumer demand for local foods, retailers were offered an easy way to promote them. The program itself also created a consensus about what local foods were in the province, a consensus that rarely exists elsewhere in Canada and around the world. Producers has benefited from the program and should continue to trust its effectiveness.

This evaluation only offers a glimpse of what the market currently offers consumers in the province of Quebec. AdQ should continue to monitor its certified products’ competitiveness to better informed the public about consumer choice and local procurement possibilities.

## Figures and Tables

**Table 1:** Instructions.

Product No.	LOCAL PRODUCTS	# of Comparator Products	Local Product was *Less Expensive* than the Comparator Product (%)	Where Local more *Expensive*, on Average (NOT ADJUSTED)	Where Local *Less Expensive*, on Average (NOT ADJUSTED)	Where Local More *Expensive* (ADJUSTED)	Where Local *Less Expensive* (ADJUSTED)
Reference number for the product (eg #1, #2, etc)	The name of the local product, its size and any other identifying descriptors	The number of products to which the local product was compared on a weekly basis. For example, if this column reads as “4,” this means the local product was compared to 4 non-local products each week.	Across the six weeks of data, this column provides how often the local product was less expensive than the comparator products as a percentage. For example, if this column read as 48%, this means that 48% of the time, the local product was less expensive than the comparator product.	This column provides a measure of percentage difference. Where the local product was more expensive, relative to the comparator products, that percentage difference was averaged across the six weeks. For example, if this column reads 23%, this means that across the six weeks, on average where the local product was more expensive, it was more expensive by 23%.	This column provides a measure of percentage difference. Where the local product was less expensive relative to the comparator products, that percentage difference was averaged across the six weeks. For example, if this column reads −31%, this means that across the six weeks, on average, where the local product was less expensive it was less expensive by −31%.	This column provides a measure of percentage difference, but in this case any large price fluctuations, those more than 20%, were removed from the average to create an adjusted average.	This column provides a measure of percentage difference, but in this case any large price fluctuations, those more than 20%, were removed from the average to create an adjusted average.

**Table 2:** Milk & Milk Alternatives.

Product No.	LOCAL PRODUCTS	# of Comparator Products	Local Product Was *Less Expensive* Than the Comparator Product (%)	Where Local More *Expensive*, on Average (NOT ADJUSTED)	Where Local *Less Expensive*, on Average (NOT ADJUSTED)	Where Local More *Expensive* (ADJUSTED)	Where Local *Less Expensive* (ADJUSTED)
68	Lactantia-Lactantia 1% Milk-2 L	7	39.31%	24.11%	−16.04%	17.63%	−9.55%
69	Sealtest-1% Milk-2 L	7	69.86%	0.82%	−23.93%	0.82%	−2.43%
70	AQuebon-1% Milk-1 L	7	52.22%	0.67%	−20.09%	0.67%	−10.07%
71	AQ-Sealtest-1% Milk-1 L	7	54.73%	2.16%	−19.08%	2.16%	−8.12%
72	Natura-Organic Soy Beverage, Enriched Original-946 Ml	2	100.00%	High Price Volatility Beyond Threshold	−14.17%	High Price Volatility Beyond Threshold	−13.22%

**Table 3:** Butter.

Product No.	LOCAL PRODUCTS	# of Comparator Products	Local Product Was *Less Expensive* Than the Comparator Product (%)	Where Local More *Expensive*, on Average (NOT ADJUSTED)	Where Local *Less Expensive*, on Average (NOT ADJUSTED)	Where Local More *Expensive* (ADJUSTED)	Where Local *Less Expensive* (ADJUSTED)
75	Lactantia-Fresh Churned Butter, Salted-454 G	13	0.00%	33.21%	High Price Volatility Beyond Threshold	7.78%	High Price Volatility Beyond Threshold
76	Natrel-Unsalted Butter-454 G	13	9.96%	14.38%	−13.22%	7.43%	−13.22%

**Table 4:** Select cheese.

Product No.	LOCAL PRODUCTS	# of Comparator Products	Local Product Was *Less Expensive* Than the Comparator Product (%)	Where Local More *Expensive*, on Average (NOT ADJUSTED)	Where Local *Less Expensive*, on Average (NOT ADJUSTED)	Where Local More *Expensive* (ADJUSTED)	Where Local *Less Expensive* (ADJUSTED)
77	Laiterie-Chalifoux.-Maison Riviera’s Cheese Line-31 % Milk Fat. Mild White Cheddar Cheese Block 270 g. -	7	86.67%	6.06%	−33.17%	6.06%	−10.32%
78	Riviera-Cheddar White Cheese Block-270 G	7	70.67%	9.39%	−30.39%	9.39%	−8.06%
79	Riviera-Cheese, Curds-200 G	7	89.00%	14.99%	−28.74%	14.99%	−10.11%
80	Riviera-Flakes Parmesan Cheese-150 G	3	100.00%	High Price Volatility Beyond Threshold	−14.53%	High Price Volatility Beyond Threshold	−14.53%
81	Agropur-L’’Extra Camembert Cheese-170 G	3	54.00%	30.46%	−7.52%	12.15%	−7.52%
82	Alexis Portneuf-L’’Extra Camembert Cheese-170 G	3	47.33%	18.48%	−18.81%	7.82%	−7.66%
83	Oka-Camembert Cheese-170 G	3	47.33%	11.68%	−23.52%	4.08%	−14.53%

**Table 5:** Seafood.

Product No.	LOCAL PRODUCTS	# of Comparator Products	Local Product Was *Less Expensive* Than the Comparator Product (%)	Where Local More *Expensive*, on Average (NOT ADJUSTED)	Where Local *Less Expensive*, on Average (NOT ADJUSTED)	Where Local More *Expensive* (ADJUSTED)	Where Local *Less Expensive* (ADJUSTED)
87	Gosselin Smokehouses-Smoked Plain Salmon-1 Container (Approx. 200 G)	2	50.00%	7.87%	−38.93%	7.87%	High Price Volatility Beyond Threshold
88	Grizzly-Grizzy Salmon Tartare-150 G	3	89.00%	High Price Volatility Beyond Threshold	−6.69%	High Price Volatility Beyond Threshold	−6.69%
89	Grizzy Salmon Tartare-150 G	3	8.25%	4.45%	−4.35%	4.45%	−4.35%

**Table 6:** Meat.

Product No.	LOCAL PRODUCTS	# of Comparator Products	Local Product Was Less Expensive Than the *Comparator* Product (%)	Where Local More *Expensive*, on Average (NOT ADJUSTED)	Where Local *Less Expensive*, on Average (NOT ADJUSTED)	Where Local More *Expensive* (ADJUSTED)	Where Local *Less Expensive* (ADJUSTED)
90	Beef Quebec-Lean Ground Beef-454 G	10	50.00%	7.87%	−38.93%	7.87%	High Price Volatility Beyond Threshold
91	Exceldor-Boneless Fillet Removed Chicken Breast-2 Breasts (Approx. 450 G)	9	89.00%	High Price Volatility Beyond Threshold	−6.69%	High Price Volatility Beyond Threshold	−6.69%
92	Nagano-Boneless Center Cut Pork Chop-2 Chops (Approx. 300 G)	4	53.40%	12.84%	−16.02%	9.45%	−1.29%

**Table 7:** Apples.

	LOCAL PRODUCTS	# of Comparator Products	Local Product Was *Less Expensive* Than the Comparator Product (%)	Where Local More *Expensive*, on Average (NOT ADJUSTED)	Where Local *Less Expensive*, on Average (NOT ADJUSTED)	Where Local More *Expensive* (ADJUSTED)	Where Local *Less Expensive* (ADJUSTED)
103	Les Croquantes-Acidulated Crispy Apples-907 G	3	72.33%	12.25%	−27.93%	12.25%	High Price Volatility Beyond Threshold
104	Les Croquantes-Aromatic Crispy Apples-907 G	3	71.00%	12.26%	−27.95%	12.42%	High Price Volatility Beyond Threshold
105	Les Croquantes-Empire Sweet Crispy Apples-907 G	3	71.00%	12.33%	−27.95%	12.33%	High Price Volatility Beyond Threshold
106	Les Croquantes-Little Crispy Apples-907 G	3	72.33%	12.00%	−27.94%	12.00%	High Price Volatility Beyond Threshold

**Table 8:** Clamshell Salads, Fresh Herbs.

Product No.	LOCAL PRODUCTS	# of Comparator Products	Local Product Was *Less Expensive* Than the Comparator Product (%)	Where Local More *Expensive*, on Average (NOT ADJUSTED)	Where Local *Less Expensive*, on Average (NOT ADJUSTED)	Where Local More *Expensive* (ADJUSTED)	Where Local *Less Expensive* (ADJUSTED)
107	Fresh Attitude-Arugula Prewashed-142 G	2	40.00%	9.75%	−20.31%	4.33%	−17.31%
108	Serres Coulombe-Fresh Basil-$5.79/Ea	3	0.00%	101.85%	High Price Volatility Beyond Threshold	16.02%	High Price Volatility Beyond Threshold
109	Fresh Attitude-Baby Kale Blend Prewashed-142 G	2	100.00%	High Price Volatility Beyond Threshold	−10.41%	High Price Volatility Beyond Threshold	−10.41%
110	Fresh Attitude-Baby Kale Organic Clamshell Salad-142 G	2	0.00%	12.00%	High Price Volatility Beyond Threshold	12.00%	High Price Volatility Beyond Threshold
111	Fresh Attitude-Blend Baby Cabbage Kale-142 G	2	87.50%	High Price Volatility Beyond Threshold	−9.22%	High Price Volatility Beyond Threshold	−9.22%
112	Fresh Attitude-Duo Kale Spinach-128 G	2	100.00%	High Price Volatility Beyond Threshold	−9.09%	High Price Volatility Beyond Threshold	−9.09%
113	Fresh Attitude-Caesar Salad With Toppings Single Sized-175 G	4	35.25%	24.75%	−16.69%	High Price Volatility Beyond Threshold	−16.69%
114	Fresh Attitude-Southwest Salad With Toppings Single Sized-0.175 Kg	4	35.25%	25.00%	−17.00%	High Price Volatility Beyond Threshold	−17.00%
115	Fresh Attitude-Spring Mix Salad With Toppings Single Sized-155 G	4	35.25%	25.00%	−17.00%	High Price Volatility Beyond Threshold	−17.00%
116	Florette-Coleslaw-454 G	3	100.00%	High Price Volatility Beyond Threshold	−13.74%	High Price Volatility Beyond Threshold	−13.74%

**Table 9:** Bread.

Product No.	LOCAL PRODUCTS	# of Comparator Products	Local Product Was *Less Expensive* Than the Comparator Product (%)	Where Local More *Expensive*, on Average (NOT ADJUSTED)	Where Local *Less Expensive*, on Average (NOT ADJUSTED)	Where Local More *Expensive* (ADJUSTED)	Where Local *Less Expensive* (ADJUSTED)
118	Auger-14 Grains Bread-600 G	2	75.00%	2.57%	−10.89%	2.57%	−10.89%
119	Auger-14 Grains Bread-600 g	2	100.00%	High Price Volatility Beyond Threshold	−10.78%	High Price Volatility Beyond Threshold	−10.78%
120	Country Harvest-14 Grains Sliced Bread-600 G	2	83.33%	11.05%	−3.99%	11.05%	−3.99%
121	Country Harvest-14 Grains Whole Wheat Bread-600 G	2	100.00%	High Price Volatility Beyond Threshold	−26.10%	High Price Volatility Beyond Threshold	−18.35%
122	Pom-100% Whole Wheat-Ultra Moist-Long Bread-675 G	5	10.00%	25.28%	−2.98%	5.16%	−2.98%
123	Pom-Wheat Bread-675 G	5	15.00%	27.60%	−4.77%	1.69%	−4.77%
124	Pom-White-Sandwich-Sliced-Ultra Moelleux Bread-675 G	5	0.00%	26.95%	High Price Volatility Beyond Threshold	6.29%	High Price Volatility Beyond Threshold
125	Pom-White Ultra Moist Bread-675 G	5	15.00%	27.60%	−4.77%	1.69%	−4.77%
126	Ace-Bread, Raisin Cinnamon-675 G	5	74.25%	18.29%	−27.48%	18.29%	−17.93%
127	Gadoua-Bread, Pain De Menage Grain Loaf-675 G	5	82.00%	16.57%	−30.22%	8.52%	−10.98%
128	Gadoua-Bread, Raisin Cinnamon-675 G	5	82.00%	14.59%	−34.07%	11.60%	−14.19%
129	Gadoua-Cinnamon Raisin Bagels—6 × 90.0 G	5	69.00%	19.87%	−26.62%	6.46%	−13.67%

**Table 10:** Bagels.

Product No.	LOCAL PRODUCTS	# of Comparator Products	Local Product Was *Less Expensive* Than the Comparator Product (%)	Where Local More *Expensive*, on Average (NOT ADJUSTED)	Where Local *Less Expensive*, on Average (NOT ADJUSTED)	Where Local More *Expensive* (ADJUSTED)	Where Local *Less Expensive* (ADJUSTED)
134	St. Viateur-Bagel Sesame 6 Bagels-480 G	4	38.20%	37.56%	−7.18%	5.99%	−7.18%
135	St. Viateur-Sesame Bagels-480 G	4	0.00%	82.72%	High Price Volatility Beyond Threshold	High Price Volatility Beyond Threshold	High Price Volatility Beyond Threshold

**Table 11:** Overall results.

SECTIONS	Number of Categorie	Local More Competitive		Neutral		Comparatives More Competitive	
**Groceries**	**22**	**55%**	12	18%	4	27%	6
**Dairy products**	8	24%	2	38%	3	38%	3
Meat, fish and seafood	4	25%	1	25%	1	50%	2
Fruit and vegetables	5	20%	1	60%	3	20%	1
Bakery	3	0%	0	100%	3	0%	0
Deli	4	50%	2	25%	1	25%	1
Frozen foods	2	0%	0	50%	1	50%	1
**T** **otal**	48		18		16		14
		**70.83% (34 categories)**					

## Data Availability

Data is contained within the article.

## References

[1] (2022). Statistics Canada Publish New Data on Environmental Research. Obes. Fit. Wellness Week.

[2] Hosseini H., Jafari S.M. (2020). Introducing nano/microencapsulated bioactive ingredients for extending the shelf-life of food products. Adv. Colloid Interface Sci..

[3] No H.K., Meyers S.P., Prinyawiwatkul W., Xu Z. (2007). Applications of Chitosan for Improvement of Quality and Shelf Life of Foods: A Review. J. Food Sci..

[4] Rai M., Ingle A.P., Gupta I., Pandit R., Paralikar P., Gade A., Chaud M.V., dos Santos C.A. (2018). Smart nanopackaging for the enhancement of food shelf life. Environ. Chem. Lett..

[5] Chowdhury P., Paul S.K., Kaisar S., Moktadir M.A. (2021). COVID-19 pandemic related supply chain studies: A systematic review. Transp. Res. Part E Logist. Transp. Rev..

[6] Hobbs J.E. (2020). Food supply chains during the COVID-19 pandemic. Can. J. Agric. Econ. Can. D’agroeconomie.

[7] Clark S.J., Dittrich L.O., Law S.M., Stará D., Barták M. (2018). Food prices, taxes, and obesity in Canada and its implications for food taxation. E+M Ekon. A Manag..

[8] Woo K.Y., Lee S.K., Chan A. (2020). Food price convergence in Canada: A nonparametric nonlinear cointegration analysis. Econ. Bull..

[9] Donaher E., Lynes J. (2016). Is local produce more expensive? Challenging perceptions of price in local food systems. Local Environ..

[10] Cvijanović D., Ignjatijević S., Vapa Tankosić J., Cvijanović V. (2020). Do Local Food Products Contribute to Sustainable Economic Development?. Sustainability.

[11] Szegedyné Fricz Á., Ittzés A., Ózsvári L., Szakos D., Kasza G. (2020). Consumer perception of local food products in Hungary. Br. Food J..

[12] Miedema K. (2019). Grow small, think big: Designing a local food system for London, Ontario. URBAN Des. Int..

[13] Sumner J., McMurtry J.J., Renglich H. (2014). Leveraging the Local: Cooperative Food Systems and the Local Organic Food Co-ops Network in Ontario, Canada. J. Agric. Food Syst. Community Dev..

[14] Stevenson A.C., Kaufmann C., Colley R.C., Minaker L.M., Widener M.J., Burgoine T., Sanmartin C., Ross N.A. (2022). A pan-Canadian dataset of neighbourhood retail food environment measures using Statistics Canada’s Business Register. Health Rep..

[15] Ayres J., Bosia M.J. (2011). Beyond Global Summitry: Food Sovereignty as Localized Resistance to Globalization. Globalizations.

[16] Drolet J. (2012). Climate change, food security, and sustainable development: A study on community-based responses and adaptations in British Columbia, Canada. Community Dev..

[17] Music J., Finch E., Gone P., Toze S., Charlebois S., Mullins L. (2021). Pandemic Victory Gardens: Potential for local land use policies. Land Use Policy.

[18] Charlebois S., Somogyi S., Music J., Cunningham C. (2019). Biotechnology in food: Canadian attitudes towards genetic engineering in both plant- and animal-based foods. Br. Food J..

[19] Huddart Kennedy E., Parkins J.R., Johnston J. (2016). Food activists, consumer strategies, and the democratic imagination: Insights from eat-local movements. J. Consum. Cult..

[20] Kortright R., Wakefield S. (2011). Edible backyards: A qualitative study of household food growing and its contributions to food security. Agric. Hum. Values.

[21] Anderson C., Maher J., Wright H. (2018). Building sustainable university-based community gardens: Volunteer perceptions of enablers and barriers to engagement and benefits received from volunteering in the Moving Feast. Cogent Soc. Sci..

[22] Clendenning J., Dressler W.H., Richards C. (2015). Food justice or food sovereignty? Understanding the rise of urban food movements in the USA. Agric. Hum. Values.

[23] Charlebois S., Music J., Faires S. (2021). The Impact of COVID-19 on Canada’s Food Literacy: Results of a Cross-National Survey. Int. J. Environ. Res. Public Health.

[24] Block D.R., Chávez N., Allen E., Ramirez D. (2011). Food sovereignty, urban food access, and food activism: Contemplating the connections through examples from Chicago. Agric. Hum. Values.

[25] Heroux L. (2020). Farmers’ markets/farm stands in southern quebec and northeastern new york/vermont: Comparative marketing strategies. J. East. Townsh. Stud..

[26] Wittman H., Beckie M., Hergesheimer C. (2012). Linking Local Food Systems and the Social Economy? Future Roles for Farmers’ Markets in Alberta and British Columbia*. Rural Sociol..

[27] Noseworthy B.L., Williams P.L., Blum I., MacLeod M. (2011). The Availability and Relative Cost of Locally Produced Foods in Grocery Stores in Nova Scotia. J. Hunger Environ. Nutr..

[28] Chong K., Chong K. (2018). Reducing Costs: Shared Service Centers, Labour and the Outsourcing Rationale. Best Practice: Management Consulting and the Ethics of Financialization in China.

[29] Avery A.A., Avery D.T. (2008). The Local Organic Food Paradigm. Georg. J. Int. Aff..

[30] Markelova H., Meinzen-Dick R., Hellin J., Dohrn S. (2009). Collective action for smallholder market access. Food Policy.

[31] Rosan C., Pearsall H. (2017). Growing A Sustainable City? In The Question of Urban Agriculture.

[32] Mudu P., Marini A. (2016). Radical Urban Horticulture for Food Autonomy: Beyond the Community Gardens Experience. Antipode.

[33] Mullins L., Charlebois S., Finch E., Music J. (2021). Home Food Gardening in Canada in Response to the COVID-19 Pandemic. Sustainability.

[34] Chao C., Zhihui T., Baozhen Y. (2017). Optimization of two-stage location–routing–inventory problem with time-windows in food distribution network. Ann. Oper. Res..

[35] Grunow M., Piramuthu S. (2013). RFID in highly perishable food supply chains–Remaining shelf life to supplant expiry date?. Int. J. Prod. Econ..

[36] Khan A.S., Salah B., Zimon D., Ikram M., Khan R., Pruncu C.I. (2020). A Sustainable Distribution Design for Multi-Quality Multiple-Cold-Chain Products: An Integrated Inspection Strategies Approach. Energies.

[37] Holley R.A., Patel D. (2005). Improvement in shelf-life and safety of perishable foods by plant essential oils and smoke antimicrobials. Food Microbiol..

[38] Allouhi A., Kousksou T., Jamil A., Agrouaz Y., Bouhal T., Saidur R., Benbassou A. (2016). Performance evaluation of solar adsorption cooling systems for vaccine preservation in Sub-Saharan Africa. Appl. Energy.

[39] Fan J., Li J., Wu Y., Wang S., Zhao D. (2016). The effects of allowance price on energy demand under a personal carbon trading scheme. Appl. Energy.

[40] Mercier S., Mondor M., Villeneuve S., Marcos B. (2018). The Canadian food cold chain: A legislative, scientific, and prospective overview. Int. J. Refrig..

[41] Suhrcke M., Stuckler D., Suk J.E., Desai M., Senek M., McKee M., Tsolova S., Basu S., Abubakar I., Hunter P. (2011). The impact of economic crises on communicable disease transmission and control: A systematic review of the evidence. PLoS ONE.

[42] Kaufhold M.-A., Gizikis A., Reuter C., Habdank M., Grinko M. (2018). Avoiding chaotic use of social media before, during, and after emergencies: Design and evaluation of citizens’ guidelines. J. Contingencies Cris. Manag..

[43] Lessard M. (2019). Le calcul des aliments du parent de fait: De l’approche synchronique à l’approche étapiste. Les Cah. Droit.

[44] Pinto J., Storey M., Brousseau S., Cecchini S., Kwong A., Mohamed H., Ryan C., Weatherbee M., Fenton T. (2021). Malnutrition in older home care clients referred to dietitians: A descriptive study. Can. J. Diet. Pract. Res..

[45] Galloway T. (2017). Canada’s northern food subsidy Nutrition North Canada: A comprehensive program evaluation. Int. J. Circumpolar Health.

[46] Harris J. (2017). A Machine Learning Approach to Forecasting Consumer Food Prices. https://dalspace.library.dal.ca/bitstream/handle/10222/73170/Harris-Jay-MEC-August-2017.pdf?sequence=1&isAllowed=y.

[47] (2017). Quebec Leads in Dairy, Maple, Pigs, and Fruits, Berries and Nuts.

[48] (2009). Canada’s Dairy Industry at a Glance. https://publications.gc.ca/site/eng/9.692475/publication.html?wbdisable=true.

[49] Cloutier J. (2017). Soy Story: A Short History of Glycine Max in Canada. https://www150.statcan.gc.ca/n1/pub/21-004-x/2017001/article/14779-eng.htm.

[50] Music J., Charlebois S., Marangoni A.G., Ghazani S.M., Burgess J., Proulx A., Somogyi S., Patelli Y. (2022). Data deficits and transparency: What led to Canada’s ‘buttergate’. Trends Food Sci. Technol..

[51] Foran P. (2020). More than Half of Canadian Restaurants May Close Permanently within Months, Survey Finds. CTVNews.ca.

[52] (2020). Canada: A Quebec mining company is fined $350,000 for a violation of the Fisheries Act. Asia News Monit..

[53] Maire E., Graham N.A.J., MacNeil M.A., Lam V.W.Y., Robinson J.P.W., Cheung W.W.L., Hicks C.C. (2021). Micronutrient supply from global marine fisheries under climate change and overfishing. Curr. Biol..

